# Conserved Enzymatic Peptides in *Bitis arietans* Venom Revealed by Comparative Proteomics: Implications for Cross-Reactive Antibody Targeting

**DOI:** 10.3390/ijms27031431

**Published:** 2026-01-31

**Authors:** Kemily Stephanie de Godoi, Fernanda Calheta Vieira Portaro, Patrick Jack Spencer, Hugo Vigerelli, Wilmar Dias da Silva

**Affiliations:** 1 Immunochemistry Laboratory, Instituto Butantan, São Paulo 05503-900, Brazil; kemilysgodoi@gmail.com; 2 Laboratory of Structure and Function of Biomolecules, Instituto Butantan, São Paulo 05503-900, Brazil; fernanda.portaro@butantan.gov.br; 3 Biotechnology Center, Nuclear and Energy Research Institute (IPEN), São Paulo 05508-900, Brazil; patrickfrombrazil@gmail.com; 4 Centre of Excellence in New Target Discovery (CENTD), Instituto Butantan, São Paulo 05503-900, Brazil; hugo.barros@butantan.gov.br

**Keywords:** complementary therapy, comparative proteomics, cross-reactive antitoxins, snake envenomation, snake venom proteases, *Viperidae* venoms

## Abstract

Snakebite envenoming remains a critical public health issue, and the molecular variability of venoms limits the cross-species efficacy of conventional antivenoms. Here, we conducted a comparative proteomic analysis of *Bitis arietans* venom to identify conserved peptide regions derived from enzymatic toxins and evaluate their potential relevance for complementary immunotherapeutic applications. Enzyme-enriched venom fractions were isolated through sequential affinity and ion-exchange chromatography and were subsequently characterized using fluorogenic FRET substrates and inhibitor assays. LC–MS/MS analysis identified 1099 proteins and revealed 36 conserved peptides within snake venom metalloproteinases (SVMPs), serine proteases (SVSPs), and phospholipase A_2_ (PLA_2_), particularly located near catalytic residues and structurally essential motifs such as the HExxHxxGxxH zinc-binding site in SVMPs, the His-Asp-Ser catalytic triad in SVSPs, and the Ca^2+^-binding loop in PLA_2_, across *Viperidae* venoms. These conserved regions were also observed in homologous toxin isoforms from additional *Viperidae* genera, supporting the evolutionary conservation of key functional domains. While sequence conservation alone does not guarantee neutralization capacity, the identified regions represent strong candidates for structural epitope mapping and targeted antibody development. This study provides a peptide-level framework for advancing complementary antibody-based therapies designed to broaden cross-species toxin recognition, reduce antivenom dosage requirements, and improve clinical outcomes in snakebite envenoming.

## 1. Introduction

The *Viperidae* family comprises ~250 species worldwide [1,2,3,4], including several genera of major epidemiological relevance. The African continent harbors a highly diverse snake fauna [5], and within the *Viperidae*, the genus *Bitis* includes 17 species of snakes found in Africa and Arabia [6,7], which is particularly relevant from a medical and epidemiological perspective. Among these, *Bitis arietans*, *Bitis gabonica*, *Bitis rhinoceros*, and *Bitis nasicornis* are of notable medical importance due to their potent venoms and frequent involvement in human envenomation cases [5]. In Sub-Saharan Africa, estimates of the annual incidence of snakebite envenoming vary widely, ranging from 56 cases per 100,000 inhabitants (95% Confidence Interval [CI]: 45–68/100,000) based on hospital records to 204 cases per 100,000 inhabitants (95% CI: 172–237/100,000) according to community-based household surveys, highlighting a substantial discrepancy between reporting systems. Case fatality rates range from 2.8% to 11.6%, while the incidence of permanent sequelae is estimated at 3–5%, and amputations account for approximately 3% of reported cases [8]. The World Health Organization (WHO) further emphasizes that community-based surveys consistently reveal a much higher incidence of snakebite envenoming than that captured by healthcare facility records, underscoring significant underreporting and limited access to effective treatment in endemic regions [9].

The pathophysiology of *Bitis arietans* envenomation has become increasingly well-characterized through in vivo and in vitro studies, which have expanded our understanding of the mechanisms underlying these effects [10,11]. Clinical manifestations such as myotoxic activity [12], hemorrhagic activity [13], tissue inflammation [14] and macrophage-driven inflammatory responses [15] are triggered by specific venom enzymes that act as key determinants of toxicity [16]. A detailed understanding of venom composition is essential for improving therapeutic strategies and developing more effective antivenoms. High-resolution analytical approaches are essential for elucidating the structural complexity and intra- and interspecific variability of venom components. Advances in proteomic strategies have proven fundamental for protein characterization, enabling the identification of conserved and/or homologous peptides that may serve as molecular targets for toxin characterization and the development of improved immunotherapeutic approaches.

Antivenoms remain the only specific and effective treatment for snakebite envenoming, although their efficacy and safety vary considerably across species [17]. Despite their indispensable role, current antivenoms require significant improvements to effectively neutralize snake venom toxins. Differences in envenomation caused by snakes of the same species, influenced by dietary factors, sex, and geographical availability, further complicate antivenom development, since a single formulation may not be equally effective against all venom compositions [8]. Most antivenoms are produced in equines and contain antibodies that neutralize both toxic and non-toxic components of the venom [5], activating the human complement system [18]. Despite their effectiveness, conventional antivenom therapy often causes adverse reactions—including allergies, serum sickness, and anaphylaxis—requiring prophylactic measures [19]. Furthermore, many antibodies produced target non-relevant or non-toxic epitopes, which can reduce therapeutic efficacy; in Bothropic antivenom, up to 95% of IgGs may be non-therapeutic [20,21,22,23]. These limitations highlight the need for continued research into venom composition and immune responses to improve antivenom specificity and support the development of complementary therapeutic strategies.

In light of these limitations, complementary therapeutic approaches have been explored, including the use of monoclonal antibodies or specific antibody fragments designed to neutralize local effects that are not effectively reversed by conventional antivenom therapy. These effects, primarily induced by snake venom metalloproteinases (SVMPs), serine proteases (SVSPs), and phospholipase (PLA_2_), represent major clinical and therapeutic challenges in envenomation management [24]. The use of monoclonal antibodies as adjuncts to antivenom therapy has demonstrated promising results in neutralizing critical local toxic effects—such as hemorrhagic, myotoxic, and edematogenic activities—thereby potentially reducing tissue damage and systemic complications [24]. Recent advances in biotechnology have enabled the development of human and humanized recombinant monoclonal antibodies capable of recognizing both linear and conformational epitopes, offering improved safety, specificity, and therapeutic precision [25]. Although still in the experimental stage, these innovations highlight the value of integrating conventional antivenom therapy with modern molecular and immunological strategies to enhance treatment efficacy and minimize adverse effects associated with equine-derived antivenoms [24].

Recent studies [26] have demonstrated that purified polyclonal antibodies obtained from isolated fractions of *Bitis arietans* venom were effective in neutralizing local hemorrhagic effects in murine models, confirming these toxins as potential immunotherapeutic targets. However, the high interspecific variability among viperid venoms limits the applicability of therapies based solely on isolated fractions. Comparative proteomic analyses across different *Viperidae* species have expanded these findings by identifying structurally conserved and homologous peptides within metalloproteinases, thereby enhancing the understanding of shared antigenic recognition regions and providing a foundation for developing antibodies or antibody fragments with broad neutralizing potential. Despite the progress achieved with conventional antivenoms, their efficacy remains constrained by the high compositional variability of venoms among snake species and populations, which hinders cross-neutralization and underscores the need for broader therapeutic strategies. The search for conserved or homologous peptides among toxins from different species represents a promising approach, as these regions tend to maintain essential structural and functional features, serving as potential molecular targets for the development of complementary therapies. Nevertheless, knowledge gaps remain regarding which toxin regions are structurally conserved and immunologically relevant.

Despite recent advances in proteomic characterization of *Bitis arietans* venom and functional studies of isolated toxin fractions, it remains unclear which peptide regions are structurally conserved across *Viperidae* toxins and therefore suitable as targets for complementary antibody therapies. Identifying these conserved regions is critical to guide rational immunogen design and expand cross-neutralization beyond the species-specific scope of conventional antivenoms. In this context, the present study advances the understanding and identification of molecular targets by employing a detailed proteomic approach to identify and characterize conserved and homologous peptides derived from enzymatic toxins of *Bitis arietans* and other *Viperidae* species. This strategy provides a framework for cross-epitope recognition and supports the rational design of monoclonal antibodies or antibody fragments aimed at enhancing the neutralizing capacity of conventional antivenoms through the development of new or complementary immunotherapies.

## 2. Results

### 2.1. Fractionation and Identification of Bitis arietans Venom Toxins by Affinity Chromatography

**Figure 1:**
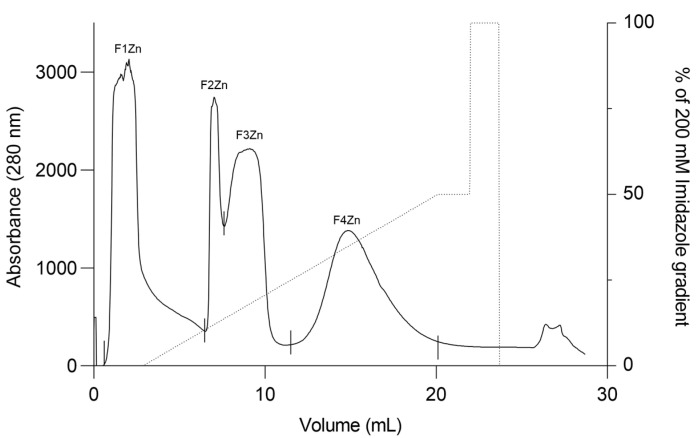
Affinity chromatography profile of *Bitis arietans* venom. Fractionation of *Bitis arietans* venom by affinity chromatography on an immobilized zinc column (HiTrap™ IMAC HP 1 mL) using 200 mM Imidazole pH 7.4. BaV (30 mg) was dispersed in 500 μL of buffer. Flow rate: 1 mL/min; fraction collected: 1 mL/tube.

**Figure 2:**
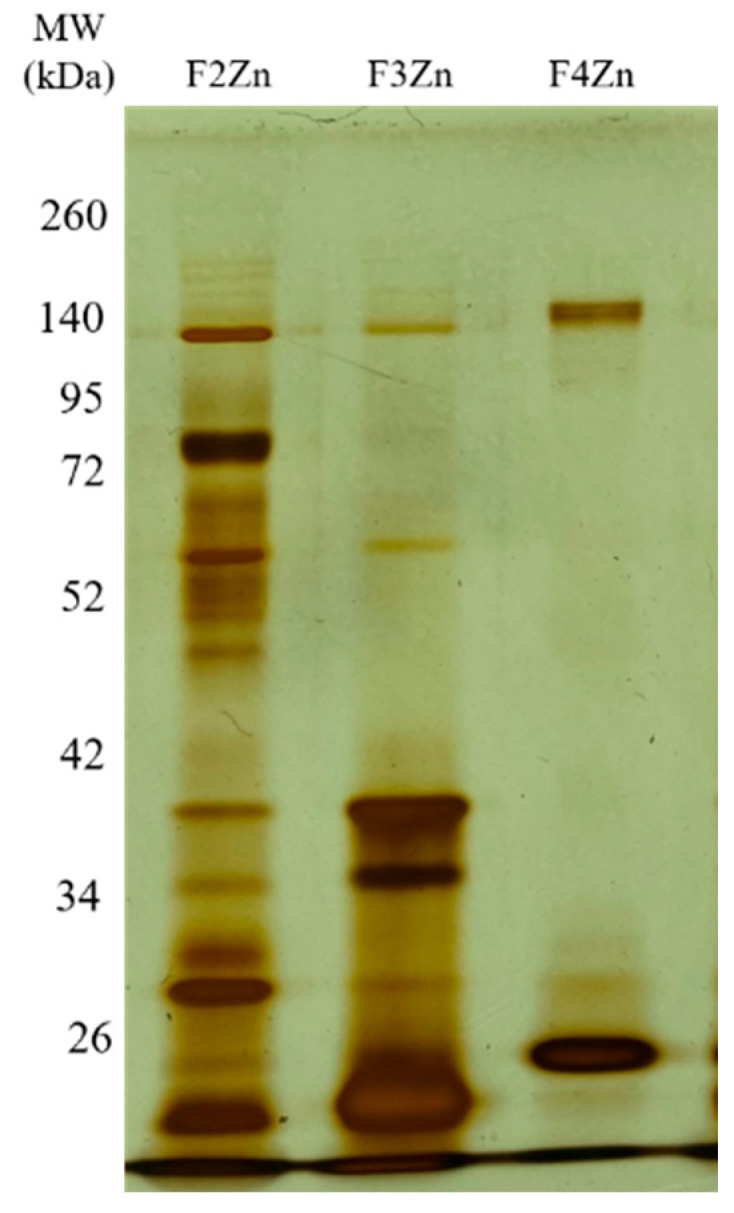
Electrophoretic profile of the *Bitis arietans* venom fractions. SDS-PAGE (under non-reducing conditions), upper gel 5%, lower gel 12.5%, stained with silver. Lanes: MW—molecular weight marker, kDa; F2Zn (fraction F2Zn); F3Zn (fraction F3Zn); and F4Zn (fraction F4Zn).

*Bitis arietans* venom (BaV) was fractionated using an immobilized zinc affinity chromatography column, resulting in the elution of four chromatographic peaks (F1Zn–F4Zn) (Figure 1). The F1Zn fraction was additionally subjected to a Benzamidine affinity column with the aim of isolating serine proteases. However, the material obtained from this complementary fractionation is part of an ongoing study and was therefore not included in the comparative analyses presented here. Thus, the structural and functional characterization described in this study focuses specifically on fractions F2Zn, F3Zn, and F4Zn, which exhibited the highest proteolytic activity associated with snake venom metalloproteinases (SVMPs) and serine proteases (SVSPs), aligning with the central objective of the present work. The SDS-PAGE profiles of the F2Zn, F3Zn, and F4Zn fractions (Figure 2) showed predominant bands in the ~25–60 kDa range, consistent with SVMPs and SVSPs. F2Zn and F3Zn displayed more intense bands around ~30–35 kDa, in agreement with their higher proteolytic activity observed in subsequent assays. In contrast, F4Zn presented fewer and less intense bands, indicating a lower abundance of enzymatic toxins.

The protein content of the fractions was determined using the bicinchoninic acid (BCA) assay, and the proteolytic activity was evaluated using the fluorogenic FRET substrates Abz-RPPGFSPFR and Abz-FRSSRQ (Table 1). As shown in Table 1 and Figure S1, the F2Zn, F3Zn, and F4Zn fractions exhibited high proteolytic activity toward the Abz-RPPGFSPFR substrate, whereas cleavage of the Abz-FRSSRQ substrate was less prominent. These results demonstrate substrate selectivity, with a clear preference for hydrolysis of the Abz-RPPGFSPFR peptide.

**Figure 3:**
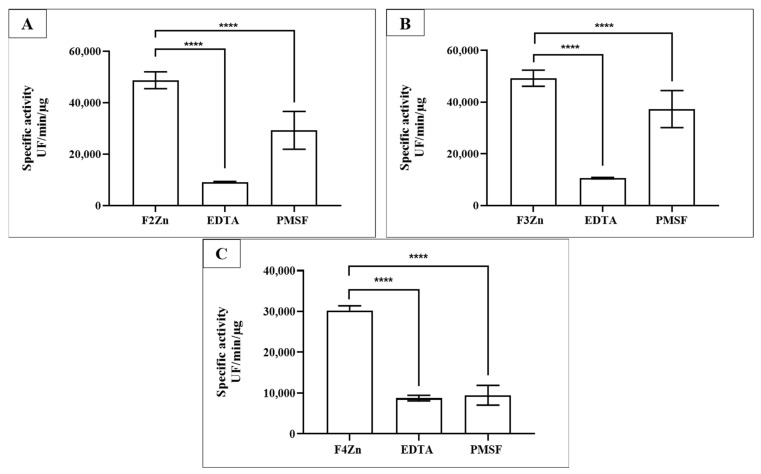
Selective inhibition of BaV fractions. Fractions obtained via affinity chromatography (F2Zn–F4Zn) were assayed for proteolytic activity against the FRET substrate Abz-RPPGFSPFR (S1). Fractions (2–4 µg/well) were pre-incubated with PMSF (2 mM/well) for 30 min at room temperature. EDTA (100 mM/well) was added immediately before measurement. Proteolysis activity was determined via spectrofluorimetry (λEX = 320 and λEM = 420 nm). (**A**) F2Zn + S1; (**B**) F3Zn + S1; (**C**) F4Zn + S1. Data are presented as mean ± SD of duplicates and are representative of two independent experiments. Statistical analysis was performed using One-Way ANOVA followed by Tukey’s test. UF: Arbitrary fluorescence unit. **** *p* < 0.0001.

Selective inhibition assays of BaV fractions were performed using Ethylenediaminetetraacetic acid (EDTA) and Phenylmethylsulfonyl fluoride (PMSF) (Figure 3). EDTA, a metal-chelating agent that inhibits SVMPs, and PMSF, an inhibitor of SVSPs, confirmed the presence of both enzyme classes in the fractions. SVMPs were predominant in F2Zn and F3Zn (Figure 3A,B), as shown by the marked reduction in activity following EDTA treatment. In contrast, the F4Zn fraction exhibited lower overall proteolytic activity compared to the other samples (Figure 3C).

### 2.2. Second Purification of Fractions Obtained Through Affinity Chromatography

**Figure 4:**
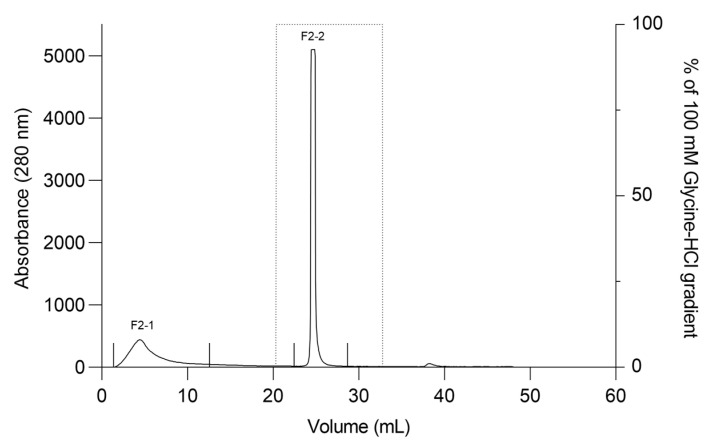
Affinity chromatography profile of F2Zn fraction. The F2Zn fraction was subjected to affinity chromatography on a Benzamidine FF (High Sub) column (HiTrap™ Benzamidine FF 1 mL), previously equilibrated with binding buffer (50 mM Tris-HCl, 1 M NaCl, pH 8.0). After sample loading, non-retained proteins were washed out, and bound material was eluted using a linear gradient of elution buffer (100 mM Glycine-HCl, pH 3.0). Chromatographic separation resulted in two main peaks (F2-1 and F2-2). Flow rate: 1 mL/min; fractions collected at 1 mL/tube.

**Figure 5:**
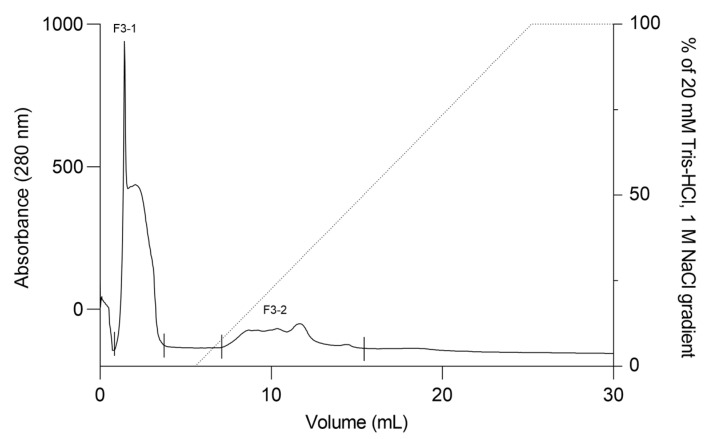
Ion exchange chromatography profile of F3Zn fraction. The F3Zn fraction was subjected to ion exchange chromatography on a Mono Q™ 5/50 GL column. The column was equilibrated with Buffer A (20 mM Tris-HCl, pH 8.0), and elution was performed using a linear gradient of Buffer B (20 mM Tris-HCl, 1 M NaCl, pH 8.0). Two major peaks were obtained, designated F3-1 and F3-2. Flow rate: 2 mL/min; fractions collected at 2 mL/tube.

**Figure 6:**
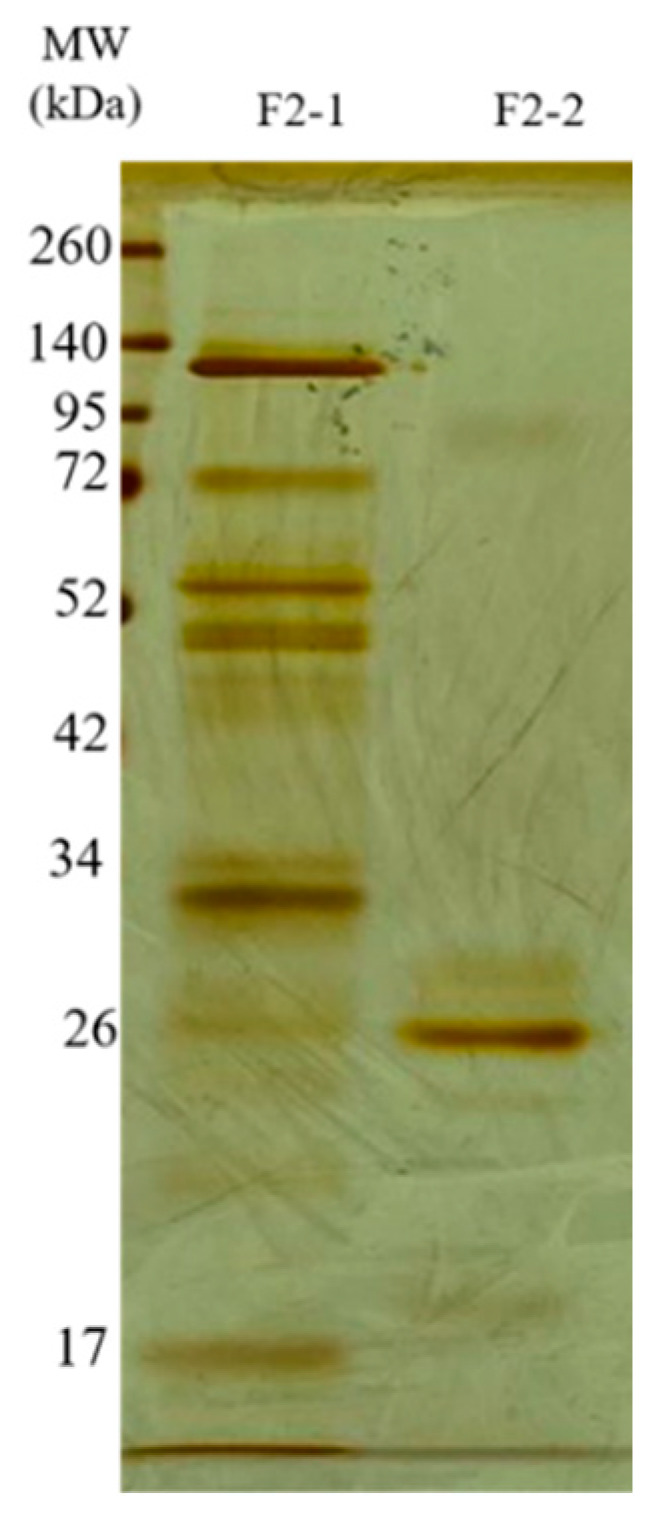
Electrophoretic profile of the F2Zn subfractions. SDS-PAGE (under non-reducing conditions), upper gel 5%, lower gel 12.5%, stained with silver. Lanes: MW—molecular weight marker, kDa; F2-1 (fraction F2-1); F2-2 (fraction F2-2).

**Figure 7:**
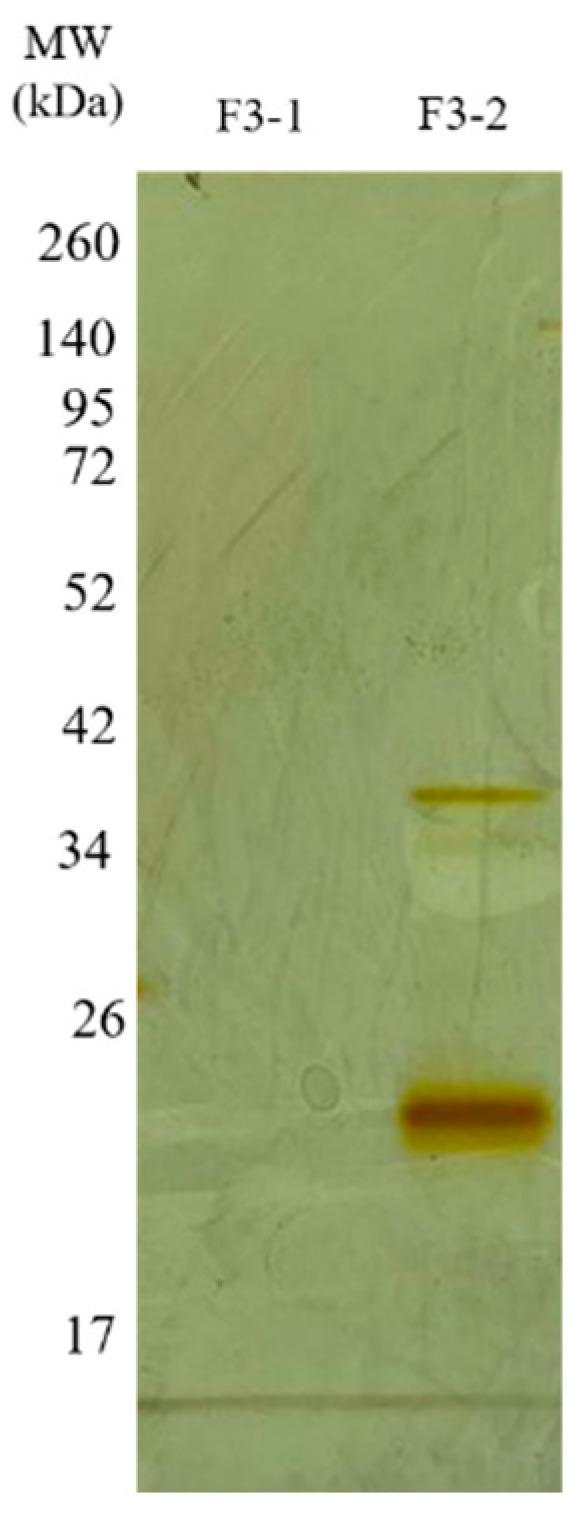
Electrophoretic profile of the F3Zn subfractions. SDS-PAGE (under non-reducing conditions), upper gel 5%, lower gel 12.5%, stained with silver. Lanes: MW—molecular weight marker, kDa; F3-1 (fraction F2-1); F3-2 (fraction F2-2).

The F2Zn and F3Zn fractions were further purified using Benzamidine FF(H)S and Mono Q™ 5/50 GL columns (Figure 4 and Figure 5), with the objective of reducing the amount of serine proteases and obtaining metalloproteinase-enriched subfractions. Both chromatographic steps resulted in the elution of two major peaks per fraction. The SDS-PAGE profiles of the F2Zn-derived subfractions (F2-1 and F2-2) (Figure 6) showed a redistribution of protein band intensities. F2-1 exhibited a predominant ~32–35 kDa band, suggesting enrichment of SVMPs, whereas F2-2 showed more intense bands around ~25–30 kDa, consistent with higher SVSP content. These changes indicate that the benzamidine affinity step contributed to the differential enrichment of protease classes. The subfractions F3-1 and F3-2 (Figure 7) showed reduced band diversity compared to the original F3Zn fraction. No clearly visible protein band was detected in F3-1 under the conditions used. In contrast, F3-2 displayed one intense band at approximately ~20 kDa and two faint bands in the ~35–40 kDa range, indicating improved separation of protein components by the ion-exchange step and enrichment of discrete subfractions.

The protein content of the subfractions was quantified using the BCA assay, and proteolytic activity was assessed using the fluorogenic substrates Abz-RPPGFSPFR and Abz-FRSSRQ (Table 2). As shown in Table 2, the F2-1 subfraction exhibited proteolytic activity against both substrates, while F2-2 showed reduced or absent activity toward Abz-FRSSRQ. The F3-1 and F3-2 subfractions demonstrated high proteolytic activity toward Abz-RPPGFSPFR, whereas cleavage of Abz-FRSSRQ was less pronounced. These data indicate substrate selectivity among the isolated subfractions, with a predominant preference for cleavage of the Abz-RPPGFSPFR peptide.

### 2.3. Identification of Protease Peptides by Mass Spectrometry Analysis

Bands of the fractions obtained from the second fractionation steps described above, were identified (Figures S2 and S3), excised and subjected to trypsin digestion and identified via mass spectrometry (LC-MS/MS). A total of 1099 proteins were identified, including 36 conserved peptides shared among *Viperidae* and *Elapidae* venoms, mainly associated with major enzymatic toxin classes. The hydrolyzed fragments were sequenced, the data processed in PEAKS DB and the sequences were subjected to bioinformatics analysis using BLASTP 2.13.0 UniprotKB/SwissProt, and the Snakes database (tax id: 8570).

The proteomic analysis resulted in the identification of peptides derived from enzymatic toxins belonging to the three main toxin classes found in *Viperidae* venoms: phospholipases A_2_ (PLA_2_s), SVMPs, and SVSPs. These peptides are listed in Table S1, along with the respective genera of snakes in which these toxins have been previously described. Homologous peptides identified in snake species from the *Elapidae* family, primarily related to Group I phospholipases A_2_, were listed separately in Table S2. These peptides were retained in the comparative analysis because they are derived from functionally active secreted toxins in venom glands. Additionally, peptides derived from non-enzymatic toxins were identified, including C-type lectins, disintegrins, and cysteine-rich secretory proteins (CRISPs), which are presented in Table S3.

### 2.4. Identification and Comparative Analyses of the Main Enzymatic Classes

**Figure 8:**
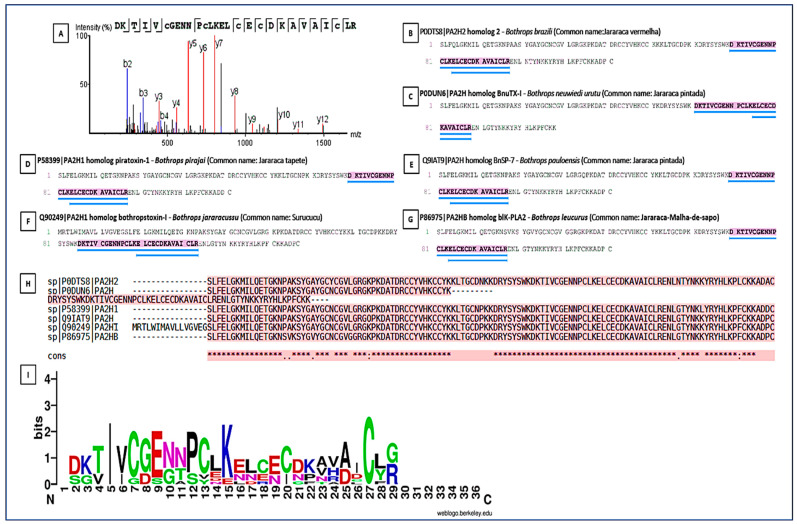
Peptide sequencing and homology in snake phospholipases. Peptide sequencing and its presence in various phospholipases from different species. (**A**) Conserved peptide sequence identified by LC–MS/MS and shared among PLA_2_s from different snake species; (**B**–**G**) Representative PLA_2_ homologs from *Bothrops* species showing the presence of the conserved peptide region. (**B**) Homolog of PLA_2_ from *Bothrops pirajai*; (**C**) Homolog of PLA_2_ from *Bothrops neuwied*; (**D**) Homolog of PLA_2_ from *Bothrops pirajai*; (**E**) Homolog of PLA_2_ from *Bothrops pauloensis*; (**F**) Homolog of PLA_2_ from *Bothrops jararacussu*; (**G**) Homolog of PLA_2_ from *Bothrops leucurus*; and (**H**) Multiple sequence alignment of PLA_2_ homologs, with the conserved peptide motif highlighted and underlined in blue. (**I**) Sequence logo representation of the conserved PLA_2_ peptide region. Letter height indicates information content (bits), reflecting residue conservation at each position. Colors represent amino acid chemical properties: acidic residues (red), basic residues including arginine (R) and lysine (K) (blue), polar residues (green), hydrophobic residues (purple), and cysteine residues (black/green). Conserved cysteine and acidic residues define the Ca^2+^-binding loop and the disulfide-stabilized catalytic core characteristic of Asp49 PLA_2_s. Numbers indicate relative amino acid positions within the aligned peptide region. An asterisk (*) denotes fully conserved amino acids, and dots (.) indicate residues with similar physicochemical properties.

PLA_2_s are essential for the local and systemic toxicity observed in envenomation. In this analysis, the fragment DKTIVCGENNPCLKELCECDKAVAICLR showed identity with PLA_2_s present in the venoms of *Bothrops* spp. (*B. pirajai*, *B. leucurus*, *B. jararacussu*, *B. brazili*, *B. neuwiedi*, *B. pauloensis*), highlighting the structural conservation of PLA_2_s in viperids (Figure 8). The sequence conservation suggests that these isoforms possibly share similar mechanisms of action typical of PLA_2_s (myotoxicity, neurotoxicity, and anticoagulant activity). The Multiple Sequence Alignment revealed the presence of a key conserved peptide motif [27], indicating a common structural region or active site, maintained throughout the evolution of PLA_2_s isoforms. This motif may be a potential target for the development of specific inhibitors. The PLA_2_s logo highlighted a highly conserved segment (VCGENNPCLKELCEC) spanning positions 6–21, representing the Ca^2+^-binding loop and disulfide-stabilized catalytic core of Asp49-type PLA_2_s. Cysteine residues are dominant across the alignment, confirming the dense disulfide network typical of this family. N- and C-terminal positions showed greater variability, corresponding to surface-exposed or flexible regions.

**Figure 9:**
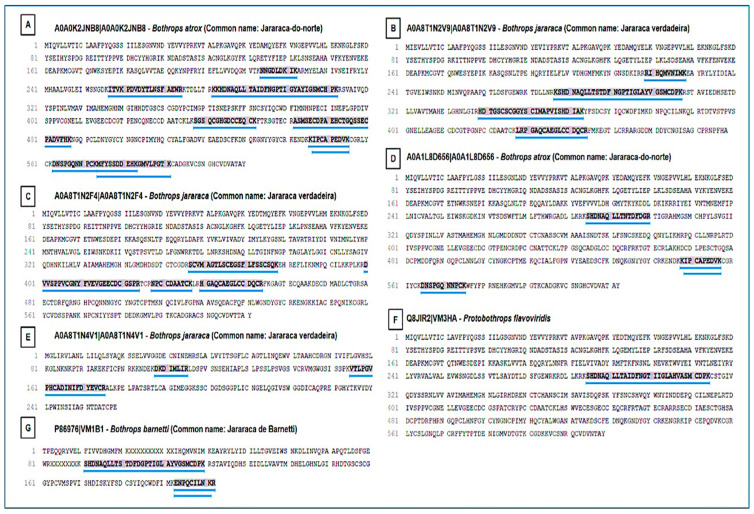
Peptide identification in snake metalloproteinases. (**A**–**G**) Representative SVMP homologs from different snake species showing peptides identified by LC–MS/MS. (**A**) Homolog of SVMP from *Bothrops atrox*; (**B**) Homolog of SVMP from *Bothrops jararaca*; (**C**) Homolog of SVMP from *Bothrops jararaca*; (**D**) Homolog of SVMP from *Bothrops atrox*; (**E**) Homolog of SVMP from *Bothrops jararaca*; (**F**) Homolog of SVMP from *Protobothrops flavoviridis*; and (**G**) Homolog of SVMP from *Bothrops barnetti*. Purple highlights indicate conserved peptide sequences detected across multiple SVMP isoforms. Blue underlines mark the conserved catalytic motif corresponding to the Zn^2+^-binding active site characteristic of snake venom metalloproteinases. Numbers indicate relative amino acid positions within the aligned peptide region. Sequence homology across species supports the evolutionary conservation of structurally and functionally essential SVMP regions.

**Figure 10:**
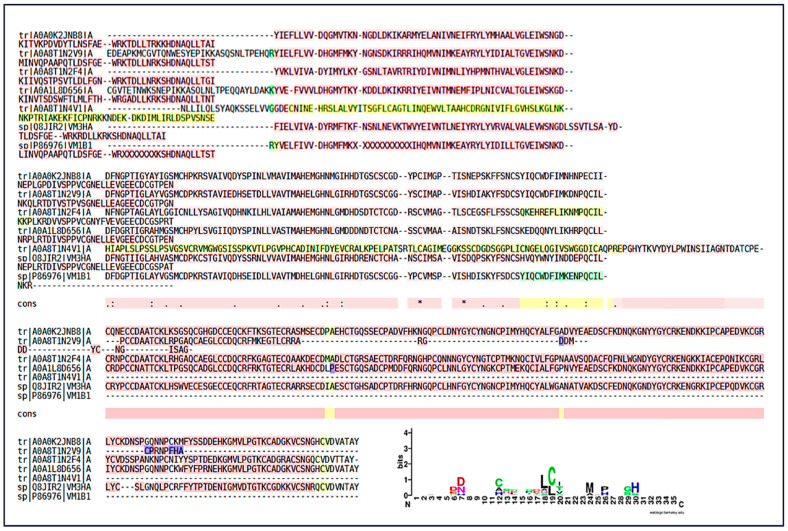
Multiple sequence alignment and conserved motifs of snake venom metalloproteinases (SVMPs). Multiple sequence alignment highlighting a conserved peptide region identified across SVMP homologs. Yellow highlighted regions indicate peptide sequences identified by LC–MS/MS and selected for comparative analysis. Conserved residues are indicated by symbols, where an asterisk (*) denotes fully conserved amino acids, and dots (.) indicate residues with similar physicochemical properties. The dotted line highlights the conserved Zn^2+^-binding catalytic motif characteristic of SVMPs. Numbers indicate relative amino acid positions within the aligned peptide region. The sequence logo summarizes residue conservation, where letter height represents information content (bits). Colors denote amino acid chemical properties: acidic residues (red), basic residues including arginine (R) and lysine (K) (blue), polar residues (green), hydrophobic residues (purple), and cysteine residues (black/green).

SVMPs were identified as one of the most abundant classes of toxins. The analysis revealed SVMPs with homology to snake metalloproteinases from *Bothrops* spp. (*B. atrox*, *B. jararaca*, *B. barnetti*) and *Protobothrops* (Figure 9). The presence of multiple entries for *Bothrops jararaca* reflects the diversity of SVMP isoforms found in the venom, each with potential functional differences. The Multiple Sequence Alignment confirmed the presence of a highly conserved peptide motif (Figure 10). This motif corresponds to the catalytic active site region of the enzyme, which is crucial for its biological function. The conservation of this sequence demonstrates the strong evolutionary pressure to maintain the capacity of SVMPs to exert their hemorrhagic, extracellular matrix-degrading, and procoagulant activities, despite species diversification. The SVMP-derived peptides displayed strong conservation of acidic residues [DE] and histidines (H) around positions 6–8 and 12–20. These residues correspond to the canonical Zn^2+^-binding catalytic motif (HEXXHXXGXXH) typical of metalloproteinases. Additional conserved cysteine (C) residues indicate structural stabilization by disulfide bridges.

**Figure 11:**
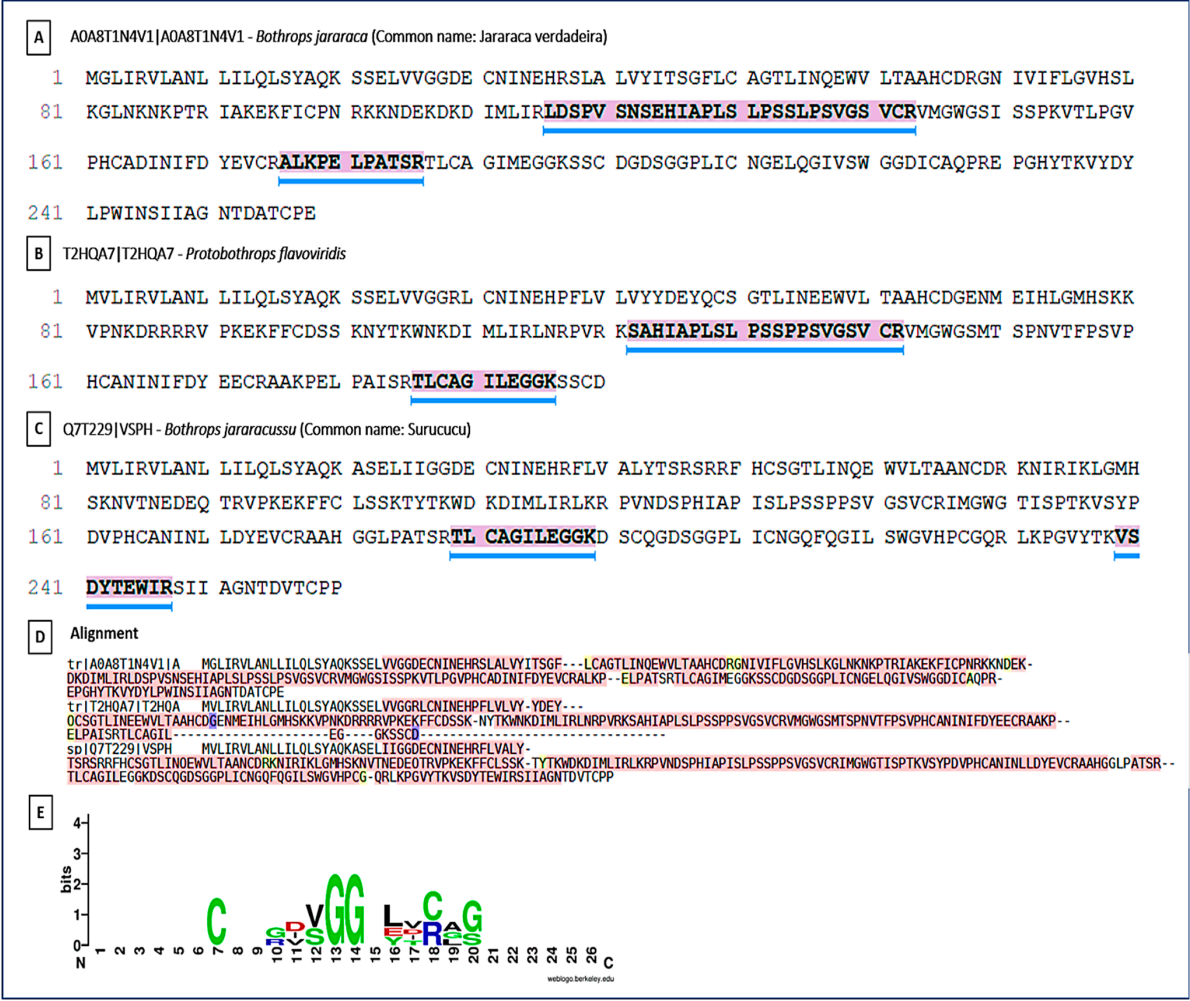
Peptide identification and sequence conservation of snake venom serine proteases (SVSPs). (**A**–**C**) Representative SVSP homologs from different snake species showing peptides identified by LC–MS/MS. Yellow highlighted regions indicate conserved peptide sequences shared among SVSPs. (**A**) Homolog of SVSP from *Bothrops jararaca*; (**B**) Homolog of SVSP from *Protobothrops flavoviridis*; (**C**) Homolog of SVSP from *Bothrops jararacussu*; and (**D**) Multiple sequence alignment highlighting conserved functional regions, with underlined residues indicating conserved motifs associated with the catalytic site. The dotted line marks the region encompassing the catalyticserine residue characteristic of trypsin-like serine proteases. Numbers indicate relative amino acid positions within the aligned peptide region. (**E**) Sequence logo representation summarizing residue conservation across SVSPs, where letter height reflects information content (bits). Colors denote amino acid chemical properties: acidic residues (red), basic residues including arginine (R) and lysine (K) (blue), polar residues (green), hydrophobic residues (purple), and cysteine residues (black/green).

Similar to SVMPs, SVSPs were prominent components in the proteomic analyses. Sequence homology was identified with SVSPs from *Bothrops* spp. (*B. jararaca*, *B. jararacussu*) and *Protobothrops* (Figure 11). The Multiple Sequence Alignment followed the same pattern of structural conservation. Although the details of the conserved motif are not evident, the identification of this motif (possibly a region of the Ser-His-Asp catalytic triad) is fundamental. The high conservation of these regions indicates that the venom maintains the structural integrity and main function of these proteases on hemostasis, interfering with the coagulation cascade through thrombin-like or fibrinogenolytic-like activities. The similarity with SVSPs from other species (such as *Protobothrops*) reinforces the principle of conservation of toxic function across the *Viperidae* family’s evolution. The SVSP logo showed conserved glycine (G) residues at positions 13–14 forming the GG loop, which is essential for substrate binding and flexibility. The LCAG segment between positions 17–20 corresponds to the loop harboring the catalytic Ser195 in the full-length enzymes. Overall, the pattern confirms the preservation of the His–Asp–Ser catalytic triad characteristic of trypsin-like SVSPs.

## 3. Discussion

In this study, we conducted a comparative proteomic analysis of *Bitis arietans* (BaV) venom fractions to identify conserved peptides derived from enzymatic toxins and assess their potential immunotherapeutic relevance. BaV venom contains a diverse repertoire of enzymes that disrupt prey hemostasis [10,28,29,30,31,32]. Conserved peptide regions were identified within snake venom metalloproteinases (SVMPs), serine proteases (SVSPs), and phospholipase A_2_ (PLA_2_), revealing structurally and functionally preserved motifs across *Viperidae* genera and highlighting their relevance as potential targets for cross-reactive antibody-based complementary therapies. These conserved motifs were located adjacent to or overlapping catalytic and metal-coordination residues, consistent with strong evolutionary constraints and supporting their relevance as antigenic determinants.

Peptides assigned to *Colubridae* and *Boidae* were excluded because they primarily corresponded to non-secretory or predicted proteins without recognized toxic roles. Therefore, the comparative analysis focused on *Viperidae* and *Elapidae*, which possess well-characterized venom profiles. Additionally, recurrent peptides from non-enzymatic toxins—including C-type lectins, disintegrins, Kunitz-type inhibitors, and Cysteine-Rich Secretory Proteins (CRISPs)—were observed, reinforcing their roles as modulators of hemostasis and supporting their consideration as complementary antigenic targets. To recover enzymatically active protease fractions suitable for functional and proteomic characterization, we employed a chromatographic workflow combining IMAC-Zn^2+^, benzamidine affinity, and anion-exchange chromatography. This orthogonal strategy was specifically designed to preserve native enzymatic activity, minimizing denaturation prior to FRET-based assays. Although RP-HPLC and SDS-PAGE–based approaches are widely used in venom proteomics, they were not prioritized here because they may compromise enzymatic functionality. Fractions F2Zn, F3Zn, and F4Zn exhibited the most representative proteolytic profiles and were therefore selected for downstream analyses. Fraction F1Zn was excluded due to low protein yield and insufficient Mass Spectrometry (MS) signal, and its subfractions will be addressed in a complementary study.

Previous evidence has demonstrated that polyclonal antibodies raised against BaV protease-rich fractions can neutralize hemorrhagic activity in vivo [26]. The conserved SVMP- and PLA_2_-derived peptides identified here are consistent with studies demonstrating that epitope-focused immunization strategies can promote cross-neutralization across snake species [33,34,35,36]. Advances in recombinant monoclonal antibody technologies have shown potent neutralization of venom toxins, including SVMPs and PLA_2_s, offering enhanced specificity, defined epitope targeting, and reduced batch variability when compared to conventional antivenoms [37,38,39]. Together, these findings support a combined therapeutic framework in which broad polyclonal reactivity is complemented by monoclonal precision targeting conserved toxin regions.

Our results also emphasize that epitope exposure and structural accessibility, rather than sequence conservation alone, are critical determinants of neutralization efficiency [35,40,41]. Accordingly, future studies will focus on (i) structural mapping of conserved peptides onto three-dimensional toxin models; (ii) evaluation of solvent accessibility and conformational stability; (iii) in silico screening against the human proteome to minimize off-target recognition; and (iv) experimental validation of binding and neutralization in vitro. Notably, the conserved regions identified here differ from human metalloproteinase and serine protease motifs [41], suggesting a low risk of host cross-reactivity. Sequence logo analysis corroborated the MS/MS data, revealing strong conservation of catalytic and metal-binding motifs in SVMPs (HEXXHXXGXXH), substrate-binding loops in SVSPs (GG and LCAG motifs), and the Ca^2+^-binding loop and adjacent cysteine residues in PLA_2_s. These patterns indicate that the experimentally identified peptides originate from catalytically essential and evolutionarily constrained regions, reinforcing their biological relevance as immunotherapeutic targets.

Although *Bitis* species are phylogenetically closer to Old World vipers, the comparative proteomic analysis prioritized *Bothrops* spp. due to practical and methodological considerations. At the peptide level, robust comparative analyses depend strongly on the availability, annotation quality, and curation depth of protein sequences deposited in public databases. Currently, New World viperid venoms—particularly those from *Bothrops* species—are substantially better represented in UniProt/Swiss-Prot, enabling more reliable identification of homologous and conserved peptide regions and reducing ambiguity in peptide assignment. Importantly, the conserved motifs identified correspond to catalytically essential regions under strong evolutionary constraint and are therefore expected to be preserved across *Viperidae* irrespective of geographic origin. The limited availability of curated African viperid venom sequences represents a current limitation of this study, and future investigations incorporating expanding datasets from *Bitis*, *Echis*, and *Vipera* will be essential to further validate and extend these findings within a broader Old World phylogenetic framework.

Collectively, these findings define a targeted set of conserved enzymatic peptide regions that can be prioritized in the development of antibody-based complementary therapies to conventional antivenom. Such strategies may enable broader cross-species neutralization, reduce the required therapeutic doses, and minimize adverse reactions, contributing to the advancement of next-generation snakebite treatments.

## 4. Materials and Methods

### 4.1. Bitis arietans Venom

**Figure 12:**
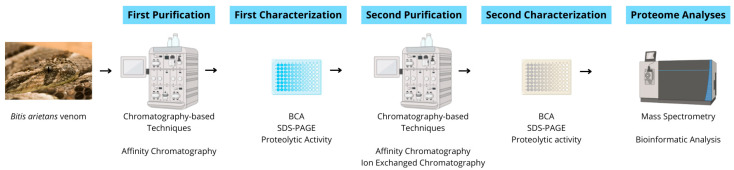
Schematic experimental workflow. Crude venom was subjected to sequential affinity and ion-exchange chromatographic steps, followed by biochemical and proteolytic characterization (Bicinchoninic Acid Assay [BCA], SDS-PAGE, and FRET-based proteolytic activity assays). Selected fractions were analyzed via LC–MS/MS for peptide and protein identification.

*Bitis arietans* venom (BaV—BA53, Venom Supplies, Tanunda, SA, Australia) was kindly donated by Venom Supplies as a commercially pooled venom batch obtained from multiple healthy adult male and female specimens originating from the African continent. The venom was supplied in lyophilized form and stored at −20 °C until use. No animals were handled directly in this study, and transport procedures complied with relevant international regulations governing the exchange of venomous animal-derived materials. All experiments were performed using the same pooled commercial venom lot. Therefore, the analyses reflect a standardized venom preparation rather than biological variability between individual specimens. Figure 12 illustrates the workflow of the experimental procedures in this study.

### 4.2. Isolation of Bitis arietans Venom Toxins by Affinity Chromatography

BaV was fractionated using a single affinity chromatography step. The venom was applied to an immobilized metal affinity chromatography column (HiTrap™ IMAC HP, Zn^2+^, 1 mL), and fraction absorbance was monitored at 280 nm using the ÄKTA Purifier system (Amersham Pharmacia Biotech AB, Uppsala, Sweden).

Approximately 30 mg of BaV was diluted in PBS pH 7.4 and loaded onto the IMAC column in a temperature-controlled room (22 ± 2 °C) at a flow rate of 1.0 mL/min. Bound proteins were eluted with 200 mM imidazole in PBS (pH 7.4), collected automatically, and concentrated to dryness using a vacuum centrifuge (SpeedVac^®^—Thermo Fisher Scientific, Waltham, MA, USA). The eluates were resuspended in sterile PBS (pH 7.4) and stored at −20 °C until further use.

### 4.3. Protein Quantification by BCA

Protein concentration was determined using the bicinchoninic acid (BCA) method with the Pierce Protein Assay kit (Thermo Fisher Scientific, Rockford, IL, USA), following the manufacturer’s instructions. A standard calibration curve was generated using bovine serum albumin (BSA—Sigma-Aldrich, St. Louis, MO, USA), and sample protein concentrations were interpolated from standard curves. Absorbance was measured at 540 nm using a microplate spectrophotometer (ELX-800—BioTek Instruments, Winooski, VT, USA). Protein concentrations of BaV and of the proteins eluted were calculated based on the BSA standard curve. Each measurement was performed in technical replicates (n = 2) obtained from the same preparation of each fraction to ensure reproducibility of the measurements.

### 4.4. Electrophoretic Profiles of the Obtained Fractions

Fractions obtained from chromatographic steps (5 μg of protein per sample) were analyzed via SDS-PAGE. Gel images were acquired and figures were assembled using Microsoft PowerPoint (version 16.0, Microsoft Corporation, Redmond, WA, USA). Gradient 5–12% polyacrylamide gels were used to improve protein band resolution. Samples were mixed with non-reducing Laemmli sample buffer in a 1:1 ratio prior to loading and electrophoresed at a constant voltage of 100 V for approximately 3 h. After electrophoresis, the gels were developed using silver nitrate staining according to Morrissey (1981) [28].

### 4.5. Proteolytic Activity of Bitis arietans Venom and Chromatographic Fractions on FRET Substrates

Proteolytic activity of the fractions (2–4 µg protein per well) was assessed in PBS (final volume 100 µL) using 96-well plates and the synthetic FRET substrates Abz-RPPGFSPFR and Abz-FRSSRQ (5 µM). Substrate hydrolysis was monitored at 37 °C in a fluorimeter (Hidex™, Turku, Finland) with excitation at 320 nm and emission at 420 nm (λEX = 320 nm; λEM = 420 nm). All activity measurements were normalized by protein mass (µg) and are reported as blank-subtracted fluorescence rates, obtained by subtracting signals from substrate-only and buffer controls. Proteolytic activity was expressed as fluorescence units per minute per microgram of protein (UF/µg/min). Assays were performed in technical duplicate (n = 2) for each purified fraction and for the crude *Bitis arietans* venom, using aliquots from the same standardized venom batch. Biological replicates were not included because the objective of this study was the proteomic and functional characterization of venom fractions rather than comparison of inter-individual or geographic variability.

### 4.6. Inhibition of Proteolytic Activities of Chromatography Fractions by Selective Inhibitors

Fractions were pre-incubated for 30 min at room temperature with the serine protease inhibitor Phenylmethylsulfonyl fluorido (PMSF) (2 mM) or PBS. The metal-chelating agent Ethylenediaminetetraacetic acid (EDTA) (100 mM) was added directly to the reaction mixture. Control samples received the corresponding buffer or ethanol used in PMSF stock preparation. Proteolytic activity was monitored using the same FRET substrates and fluorescence detection parameters described above (37 °C; λEX = 320 nm; λEM = 420 nm). Abz-RPPGFSPFR was selected as the primary substrate because it is highly susceptible to cleavage by snake venom metalloproteinases and provides higher and more consistent hydrolysis rates across venom fractions, enabling robust assessment of selective inhibition. Abz-FRSSRQ was used as a complementary substrate to evaluate substrate selectivity and the contribution of serine proteases. Activity readings were normalized by protein mass and expressed as blank-subtracted fluorescence rates to ensure that inhibitor effects reflected true enzymatic inhibition rather than differences in protein loading. Proteolytic activity was expressed as fluorescence units per minute per microgram of protein (UF/µg/min). All assays were performed in technical duplicate (n = 2) using aliquots from the same preparation of each venom fraction. Biological replicates were not included because the aim of the study was to characterize the enzymatic composition of chromatographic fractions rather than to assess venom variability.

### 4.7. Purification of Zinc Affinity Fractions by Ion Exchange and Benzamidine Affinity Chromatography

Fractions obtained from the first affinity chromatography step were screened for proteolytic activity to confirm the presence of proteases. Based on these results, fractions F2Zn and F3Zn were selected for snake venom metalloproteinase (SVMP) enrichment. Fraction F2Zn was subjected to a second affinity purification using a benzamidine affinity column (HiTrap™ Benzamidine FF (High Sub), 1 mL). Bound proteins were eluted with 100 mM glycine-HCl (pH 3) and collected across each absorbance peak. Fraction F3Zn was further purified by anion-exchange chromatography on a Mono Q™ 5/50 GL column (GE Healthcare, Uppsala, Sweden) using an ÄKTA Purifier™ system (Amersham Pharmacia Biotech AB, Uppsala, Sweden), with absorbance monitored at 280 nm. Proteins were eluted at a flow rate of 2 mL/min using a linear NaCl gradient from 0 to 1 M in 20 mM Tris-HCl buffer (pH 8.0), and fractions were automatically collected at the end of each absorbance peak. Subfractions obtained from F2Zn (F2-1 and F2-2) and F3Zn (F3-1 and F3-2) were stored at −20 °C until use. Protein concentration was determined using the bicinchoninic acid (BCA) method, and electrophoretic profiles were evaluated via SDS-PAGE. The fractions obtained from this step were subsequently evaluated for proteolytic activity to identify those enriched in SVMPs and serine proteases (SVSPs), which guided the selection of fractions for further analyses, as described in Section 4.5.

### 4.8. Identification of Protease Fragment Peptides by Mass Spectrometry Analysis

Protein search was performed using PEAKS Studio 12 software, with trypsin defined as the designated cleavage enzyme, searching against the UniprotKB/SwissProt “Serpentes” database (taxid: 8570). A single representative sample of each selected band (F2-1, F2-2, and F3-2) was analyzed via LC–MS/MS. Identifications were accepted under a 0.1% FDR threshold at both the protein and peptide levels, requiring at least one unique peptide match. Mass error tolerances were set to 20 ppm for precursor ions and 40 ppm for fragment ions. Methionine oxidation and N-terminal acetylation were defined as variable modifications, while cysteine carbamidomethylation was defined as a fixed modification. A maximum of two miscleavages were allowed under trypsin digestion conditions. Peptides were considered homologous when showing >90% sequence similarity across protein entries. When a peptide matched multiple accessions, a single preferred accession was reported, prioritized based on phylogenetic proximity within *Viperidae* (*Bitis* > *Echis* > *Vipera* > *Bothrops* > *Protobothrops* > *Lachesis* > *Gloydius* > *Sistrurus* > *Crotalus* > *Montivipera* > *Deinagkistrodon*). In cases where multiple proteins shared equivalent phylogenetic positioning, the accession with the highest −10lgP score was selected as the primary representative.

To highlight conserved amino acid motifs within the three major enzymatic toxin families, SVMPs, SVSPs, and phospholipases A_2_ (PLA_2_s), peptide sequences identified via LC–MS/MS were aligned with homologous toxin sequences retrieved from the UniProt database (Tables S1–S3). Multiple sequence alignments (MSAs) were performed using Clustal Omega (https://www.ebi.ac.uk/Tools/msa/clustalo/ (accessed on 10 October 2025) with default parameters. The resulting aligned FASTA files were then analyzed using WebLogo version 3.7 (https://weblogo.berkeley.edu/ (accessed on 10 October 2025). Sequence logos were generated with the protein option enabled, default color scheme, white background, and output resolution of 300 dpi. In each logo, the height of the letters represents the information content (bits) for each alignment position, calculated from the Shannon entropy, reflecting residue conservation. Letter color corresponds to chemical properties: acidic (red), basic (blue), polar (green), hydrophobic (orange/black), and cysteine (black/green).

### 4.9. Statistics Analysis

Data are expressed as the mean (M) ± standard deviation (SD) of technical duplicates (n = 2). As the study design involved characterization of a single standardized venom preparation, statistical comparisons reflect variation between technical replicates rather than biological replicates. Comparisons between groups were performed via One-Way ANOVA followed by Tukey’s post-test using GraphPad Prism version 8.4.3 (GraphPad Software Inc., San Diego, CA, USA).

## Figures and Tables

**Table 1:** Protein content and enzymatic activity of *Bitis arietans* fractions.

Venom and Fractions	Protein Content (µg/mL)	Specific Activities (UF/min/µg)	
		Abz-RPPGFRSPFR	Abz-FRSSRQ
BaV	1531	49,193.5 ± 4339	21,623.5 ± 4104
F2Zn	1056	48,747.3 ± 3254	4445.2 ± 297.1
F3Zn	744	49,257.3 ± 3129	5171.4 ± 505.0
F4Zn	410	30,254.4 ± 1114	4086.4 ± 122.7

Protein concentration (µg/mL) was quantified using the BCA method. Specific enzymatic activity was quantified via spectrofluorimetry (λEX = 320 and λEM = 420 nm) using the FRET substrates Abz-RPPGFSPFR and Abz-FRSSRQ at 5 µM in PBS. Results are expressed as the mean (M) ± standard deviation (SD). BaV: *Bitis arietans* venom. UF/min/µg: fluorescence units/min/µg (arbitrary units).

**Table 2:** Protein content and enzymatic activity of F2 and F3 subfractions.

Fractions	Protein Content (µg/mL)	Specific Activities (UF/min/µg)	
		Abz-RPPGFRSPFR	Abz-FRSSRQ
F2-1	148	69,324.9 ± 1559	49,089.8 ± 5907
F2-2	69	26,096.8 ± 4380	3634.2 ± 90.46
F3-1	22	56,922.3 ± 8473	4825.2 ± 589.5
F3-2	84	63,065.6 ± 1344	3824.07 ± 246.1

Protein concentration (µg/mL) was determined using the BCA assay. Specific enzymatic activity was measured via spectrofluorometry (λEX = 320 and λEM = 420 nm) using the FRET substrates Abz-RPPGFSPFR and Abz-FRSSRQ at 5 µM in PBS. Results are expressed as the mean (M) ± standard deviation (SD). UF/min/µg: fluorescence units/min/µg (arbitrary units).

## Data Availability

The raw data supporting the conclusions of this article will be made available by the authors on request.

## Supplementary Materials

The following supporting information can be downloaded at: https://www.mdpi.com/article/10.3390/ijms27031431/s1.
