# Supplementary material for: Conserved Enzymatic Peptides in Bitis arietans Venom Revealed by Comparative Proteomics: Implications for Cross-Reactive Antibody Targeting
Source: Int J Mol Sci. 2026 Jan 31;27(3):1431. doi: 10.3390/ijms27031431 (PMC12898025; doi:10.3390/ijms27031431)
Supplement: Supplementary file 1 [file ijms-27-01431-s001.zip › Supplementary material 1 - Figure S1.pdf]

Figure S1. Proteolytic activity of *Bitis arietans* venom and derived chromatographic fractions measured using FRET substrates.

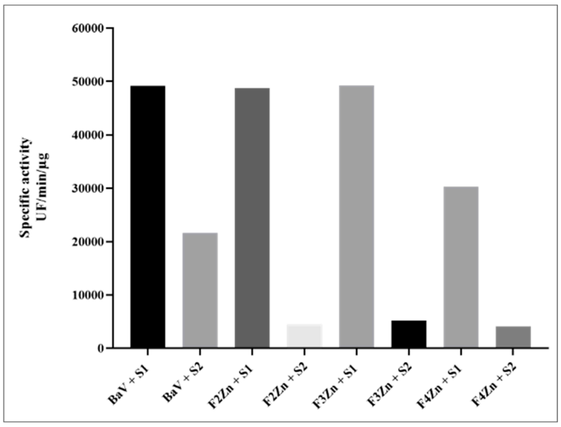

Specific enzymatic activity was measured by spectrofluorimetry (λEX = 320 nm; λEM = 420 nm) using the FRET substrates Abz-RPPGFSPFR and Abz-FRSSRQ at 5 µM in PBS (final volume 100 µL). Results are expressed as mean (M) ± standard deviation (SD) of technical duplicates. BaV: *Bitis arietans* venom; UF/min/µg: fluorescence units/min/µg (arbitrary units).
