# Supplementary material for: Conserved Enzymatic Peptides in Bitis arietans Venom Revealed by Comparative Proteomics: Implications for Cross-Reactive Antibody Targeting
Source: Int J Mol Sci. 2026 Jan 31;27(3):1431. doi: 10.3390/ijms27031431 (PMC12898025; doi:10.3390/ijms27031431)
Supplement: Supplementary file 1 [file ijms-27-01431-s001.zip › Supplementary material 3 - Figure S3.pdf]

Figure S3. Electrophoretic profile of the subfraction F3-2 obtained from the F3Zn fraction.

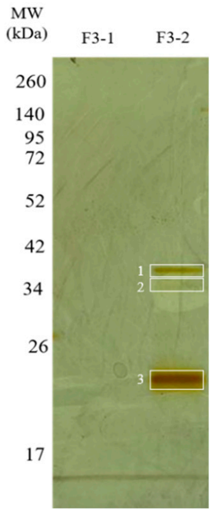

SDS-PAGE was performed under non-reducing conditions using a 5% stacking gel and a 12.5% resolving gel, followed by silver staining. Lanes: MW – molecular weight marker (kDa); F3-1 – subfraction F3-1; F3-2 – subfraction F3-2. Numbered bands indicate the gel regions excised for identification by LC–MS/MS analysis.
