# Supplementary material for: Conserved Enzymatic Peptides in Bitis arietans Venom Revealed by Comparative Proteomics: Implications for Cross-Reactive Antibody Targeting
Source: Int J Mol Sci. 2026 Jan 31;27(3):1431. doi: 10.3390/ijms27031431 (PMC12898025; doi:10.3390/ijms27031431)
Supplement: Supplementary file 1 [file ijms-27-01431-s001.zip › Supplementary material 4 - Table S1.pdf]

Table S1. Protein identification and snake genus classification among *Viperidae* venoms.

Peptide ID	Peptide sequence	Class	Subclass	Homologous species	Region type	Found in fractions	Protein accession
P0001	STAVIEDHSETDLLVAVTMAHELGHNLGIR	SVMP	P-I	*Bothrops*	SVMP core region	F2-2:1	A0A8T1N161
P0002	RSTAVIEDHSETDLLVAVTMAHELGHNLGIR	SVMP	P-I	*Bothrops*	SVMP core region	F2-2:1	A0A8T1N161
P0003	HDTGSCSCGGYSCIMAPVISHDIAK	SVMP	P-I	*Bothrops*	Cysteine-rich domain (CRD, structural)	F2-2:1; F3-2:2	A0A8T1N161
P0004	DKTIVCGENNPCLKELCECDKAVAICLR	PLA_2	Basic PLA_2	*Bothrops*	PLA_2 structural core	F2-2:1	P0DUN6
P0005	ELCECDKAVAICLR	PLA_2	Basic PLA_2	*Bothrops, Lachesis*	PLA_2 structural core	F2-2:1	P0DUN6
P0006	DKTIVCGENNSCLKELCECDK	PLA_2	Basic PLA_2	*Bothrops, Lachesis*	PLA_2 structural core	F2-2:1	P86453
P0007	DKTIVCGENNSCLKELCECDKAVAICLR	PLA_2	Basic PLA_2	*Bothrops, Lachesis*	PLA_2 structural core	F2-2:1	P86453

P0008	KKHDNAQLLTAIDFNGPTIGYAYIGSMCHPK	SVMP	P-I	*Bothrops*	SVMP core region	F2-2:1; F3-2:2	A0A8T1N174
P0009	ASMSECDPAEHCTGQSSECPADVFHK	SVMP	P-I	*Bothrops, Crotalus, Deinagkistrodon, Sistrurus*	Cysteine-rich domain (CRD, structural)	F2-2:1; F2-2:4; F3-2:1; F3-2:2	A0A8T1N174
P0010	VTLPGVPHCADINIFDYEVCR	SVSP	-	*Bothrops*	Surface / substrate-binding loop (SVSP)	F2-1:1; F2-2:1; F3-2:2	A0A8T1N4V1
P0011	DVVSPPVCGNYFVEVGEECDCGSPR	SVMP	P-I	*Bothrops*	Cysteine-rich domain (CRD, structural)	F2-2:1; F3-2:2	A0A8T1N2F4
P0012	YIELAVVADHGMFTK	SVMP	P-I	*Bothrops*	SVMP core region	F2-2:1	A0A8T1N4P7
P0013	SECDIAESCTGQSAECPTDNFER	SVMP	P-I	*Bothrops*	Cysteine-rich domain (CRD, structural)	F2-2:1	A0A8T1N169
P0014	IYETVNALNVICR	SVMP	P-I	*Bothrops*	SVMP core region	F2-2:1	A0A8T1N447

P0015	SGYITCGK	PLA_2	Basic PLA_2	*Crotalus*	PLA_2 structural core	F2-2:2	P86169
P0016	HLLQFNKM	PLA_2	Basic PLA_2	*Crotalus*	PLA_2 structural core	F2-2:2	P86169
P0017	SGYLTCGK	PLA_2	Basic PLA_2	*Lachesis*	PLA_2 structural core	F2-2:2	P0C943
P0018	VSLVNKNDDTCTGQSADCPR	SVMP	P-II	*Crotalus*	Cysteine-rich domain (CRD, structural)	F2-2:2	Q2QA03
P0019	IPCAPQDVK	SVMP	P-I	*Bothrops, Crotalus, Echis, Vipera*	SVMP core region	F2-2:3	E9JG47
P0020	LRPGAQCAEGLCCDQCR	SVMP	P-I	*Bothrops, Sistrurus*	Cysteine-rich domain (CRD, structural)	F2-2:4; F3-2:2	A0A8T1N161
P0021	LYCKDNSPGQNNPCK	SVMP	P-I	*Bothrops, Crotalus, Gloydius, Sistrurus*	Cysteine-rich domain (CRD, structural)	F2-2:4	A0A8T1N174
P0022	KIPCAPEDVK	SVMP	P-I	*Bothrops, Echis, Gloydius, Sistrurus*	cysteine-rich domain	F2-1:2; F2-2:5; F3-2:2	A0A0A1WDV1

					(CRD, structural)		
P0023	IPCAPEDVK	SVMP	P-I	*Bothrops, Echis, Gloydius, Sistrurus*	SVMP core region	F2-1:2; F2-2:5; F3-2:2	A0A0A1WDV1
P0024	NNGDLDKIK	SVMP	P-I	*Bothrops*	SVMP core region	F2-2:5; F3-2:2	A0A8T1N174
P0025	KTDLLTR	SVMP	P-I	*Bothrops*	SVMP core region	F2-1:2; F2-2:5	A0A8T1N174
P0026	IYEIVNFLNEIFR	SVMP	P-I	*Bothrops*	SVMP core region	F2-1:1	A0A8T1N4S1
P0033	LDSPVSNSEHIAPLSLPSSPPSVGSVCR	SVSP	Thrombin-like	*Crotalus*	Catalytic triad vicinity (active site)	F2-1:2	A0A0U2UH64
P0034	VLNEDEQTR	SVSP	Thrombin-like	*Bothrops, Crotalus, Deinagkistrodon*	Surface / substrate-binding loop (SVSP)	F2-1:2	Q072L6
P0035	KVLNEDEQTR	SVSP	Thrombin-like	*Bothrops, Crotalus, Deinagkistrodon*	Surface / substrate-binding loop (SVSP)	F2-1:2	Q072L6

P0036	VSNSEHIAPLSLPSSPPSVGSVCR	SVSP	Thrombin-like	*Bothrops*	Surface / substrate-binding loop (SVSP)	F2-1:2	Q072L6
P0042	LDKPISNSKHIAPLSLPSSPPSVGSVCR	SVSP	Thrombin-like	*Deinagkistrodon*	Catalytic triad vicinity (active site)	F2-1:2	Q9YGS1
P0043	LDSPVSNSEHIAPLSLPSSLPSVGSVCR	SVSP	Thrombin-like	*Bothrops*	Catalytic triad vicinity (active site)	F2-1:2	A0A8T1N4V1
P0044	ALKPELPATSR	SVSP	Thrombin-like	*Bothrops*	Surface / substrate-binding loop (SVSP)	F2-1:2	A0A8T1N4V1
P0045	NSAHIAPISLPSSPPIVGSVCR	SVSP	Thrombin-like	*Bothrops*	Surface / substrate-binding loop (SVSP)	F2-1:2	O13069
P0046	TLCAGILQGGK	SVSP	Thrombin-like	*Bothrops*	Surface / substrate-binding loop (SVSP)	F2-1:2	O13069

P0047	IVGGDECNINEHRF	SVSP	Thrombin-like	*Lachesis*	Surface / substrate-binding loop (SVSP)	F2-1:2	C0HLA2
P0048	SAHIAPLSLPSSPPSVGSVCR	SVSP	Thrombin-like	*Protobothrops*	Surface / substrate-binding loop (SVSP)	F2-1:2	P84787
P0049	TLCAGILEGGK	SVSP	Thrombin-like	*Bothrops, Protobothrops*	Surface / substrate-binding loop (SVSP)	F2-1:2	Q7T229
P0050	VSDYTEWIR	SVSP	Thrombin-like	*Bothrops*	Surface / substrate-binding loop (SVSP)	F2-1:2	Q7T229
P0051	GEHCISGPCCR	SVMP	P-I	*Montivipera*	Cysteine-rich domain (CRD, structural)	F2-1:9	A0A7R7T1Q6
P0052	RGEHCISGPCCR	SVMP	P-I	*Montivipera*	Cysteine-rich domain (CRD, structural)	F2-1:9	A0A7R7T1Q6

P0053	MFYSSDDEHKGMVLPGTK	SVMP	P-I	*Bothrops*	SVMP core region	F3-2:2	A0A0K2JNB8
P0054	ITVKPDVDYTLNSFAEWR	SVMP	P-I	*Bothrops*	SVMP core region	F3-2:2	A0A0K2JNB8
P0055	MFYSSDDEHK	SVMP	P-I	*Bothrops*	SVMP core region	F3-2:2	A0A0K2JNB8
P0056	DNSPGQNNPCK	SVMP	P-I	*Bothrops*	SVMP core region	F3-2:2	A0A0K2JNB8
P0057	KSHDNAQLLTSTDFNGPTIGLAYVGSMCDPK	SVMP	P-I	*Bothrops*	SVMP core region	F3-2:2	A0A8T1N2V9
P0058	RIHQMVNIMK	SVMP	P-I	*Bothrops*	SVMP core region	F3-2:2	A0A8T1N2V9
P0059	HGAQCAEGLCCDQCR	SVMP	P-I	*Bothrops*	Cysteine-rich domain (CRD, structural)	F3-2:2	A0A8T1N2F4
P0060	SCVMAGTLSCEGSFLFSSCSQK	SVMP	P-I	*Bothrops*	Cysteine-rich domain (CRD, structural)	F3-2:2	A0A8T1N2F4
P0061	NPCCDAATCK	SVMP	P-I	*Bothrops*	Cysteine-rich	F3-2:2	A0A8T1N2F4

					domain (CRD, structural)		
P0062	KSGVIICGEGTPCEK	PLA_2	Basic PLA_2	*Bothrops*	PLA_2 structural core	F3-2:2	A0A2H4N391
P0063	SGVIICGEGTPCEK	PLA_2	Basic PLA_2	*Bothrops*	PLA_2 structural core	F3-2:2	A0A2H4N391
P0064	CCFVHDCCYGK	PLA_2	Basic PLA_2	*Bothrops*	His-Asp catalytic dyad region	F3-2:2	A0A2H4N391
P0065	AAAVCFR	PLA_2	Basic PLA_2	*Bothrops*	PLA_2 structural core	F3-2:2	A0A2H4N391
P0066	MILEETKK	PLA_2	Basic PLA_2	*Bothrops*	PLA_2 structural core	F3-2:2	A0A2H4N391
P0067	MILQETGKNPVTSYGAYGCNCGVLGR	PLA2	Basic PLA_2	*Bothrops*	PLA_2 structural core	F3-2:2	A0A1L8D5Z7
P0068	DKTIVCDENNPCLK	PLA_2	Basic PLA_2	*Bothrops*	PLA_2 structural core	F3-2:2	A0A1L8D5Z7
P0069	AVAICLR	PLA_2	Basic PLA_2	*Bothrops*	PLA_2 structural core	F3-2:2	A0A1L8D5Z7

P0070	TIVCDENNPCLK	PLA_2	Basic PLA_2	*Bothrops*	PLA_2 structural core	F3-2:2	A0A1L8D5Z7
P0071	SHDNAQLLTNTDFDGR	SVMP	P-I	*Bothrops*	SVMP core region	F3-2:2	A0A1L8D656
P0072	LRPGQQCAEGLCCDQCR	SVMP	P-II	*Gloydius*	Cysteine-rich domain (CRD, structural)	F3-2:2	Q9YI19
P0073	SHDNAQLLTAIDFNGTIIGLAHVASMCDPK	SVMP	P-II	*Protobothrops*	SVMP core region	F3-2:2	Q8JIR2
P0074	VIGGDECNINEHRF	SVSP	Thrombin-like	*Calloselasma*	Surface / substrate-binding loop (SVSP)	F3-2:2	P26324
P0075	SHDNAQLLTSTDFDGPTIGLAYVGSMCDPK	SVMP	P-I	*Bothrops*	SVMP core region	F3-2:2	P86976
P0076	ENPQCILNK	SVMP	P-I	*Bothrops*	SVMP core region	F3-2:2	P86976

The table shows peptides identified by LC–MS/MS and mapped to venom metalloproteinases (SVMPs), serine proteases (SVSPs), and phospholipases A_2 (PLA_2) across *Viperidae* genera. For each peptide, the sequence, toxin class and subclass, homologous species, structural region, detected chromatographic fractions, and protein accessions are shown. Peptides were considered homologous when showing >90% sequence similarity. When a peptide matched multiple entries, a preferred protein accession was assigned based on phylogenetic proximity within *Viperidae* and highest −10lgP score, with all original matched accessions also provided.
