# Supplementary material for: Conserved Enzymatic Peptides in Bitis arietans Venom Revealed by Comparative Proteomics: Implications for Cross-Reactive Antibody Targeting
Source: Int J Mol Sci. 2026 Jan 31;27(3):1431. doi: 10.3390/ijms27031431 (PMC12898025; doi:10.3390/ijms27031431)
Supplement: Supplementary file 1 [file ijms-27-01431-s001.zip › Supplementary material 5 - Table S2.pdf]

Table S2. Protein identification and snake genus classification among non-*Viperidae* families.

Peptide ID	Peptide sequence	Class	Subclass	Homologous species	Region type	Found in fractions	Protein accession
P0027	APYNKNNENINPNR	PLA_2	Basic PLA_2 - Group I (*Elapidae*)	*Micrurus*	PLA_2 structural core	F2-2:1	Q8AXW7
P0028	VHDDCYGAAEKYHR	PLA_2	Basic PLA_2 - Group I (*Elapidae*)	*Micrurus*	PLA_2 structural core	F2-2:1	Q8AXW7
P0029	VHDDCYGAAEK	PLA_2	Basic PLA_2 - Group I (*Elapidae*)	*Micrurus*	PLA_2 structural core	F2-2:1	Q8AXW7
P0030	NNENINPNR	PLA_2	Basic PLA_2 - Group I (*Elapidae*)	*Micrurus*	PLA_2 structural core	F2-2:1	Q8AXW7
P0031	TAALCFGR	PLA_2	Basic PLA_2 - Group I (*Elapidae*)	*Micrurus*	PLA_2 structural core	F2-2:1	Q8AXW7
P0032	GGSGTPVDELDR	PLA_2	Basic PLA_2 -	*Micrurus*	PLA_2 structural core	F2-2:1	A0A2D4NPI3

			Group I (*Elapidae*)				
P0037	AAKDDCDLPELCTGR	SVMP	P-I	*Micrurus*	Metalloprotease fold region (non-venom role unconfirmed)	F2-2:1	A0A194AS47
P0038	NQCIALMGSGVK	SVMP	P-I	*Micrurus*	Metalloprotease fold region (non-venom role unconfirmed)	F2-2:1	A0A194AS47
P0039	RNDNAQLLTR	SVMP	P-I	*Micrurus*	Metalloprotease fold region (non-venom role unconfirmed)	F2-2:1	A0A194AS47
P0040	IDFNGNTLGLAHIGSLCSPK	SVMP	P-I	*Micrurus*	Metalloprotease fold region (non-venom role unconfirmed)	F2-2:1	A0A194AS47
P0041	DDCDLPELCTGR	SVMP	P-I	*Micrurus*	Cysteine-rich domain (CRD, structural)	F2-2:1	A0A194AS47

The table presents peptides showing sequence similarity to *Bitis arietans*–derived toxins but belonging to Group I phospholipases A_2 (PLA_2) and snake venom metalloproteinase-like (SVMP-like) proteins from *Elapidae* species. These sequences were retained for comparative evolutionary analysis due to their structural and catalytic relevance, but were not included in antigenic conservation interpretation due to uncertain toxic roles in *Elapidae* metalloproteases. Peptides were considered homologous when showing >90% sequence similarity, and preferred accessions were assigned as described for Table S1.
