# Supplementary material for: Conserved Enzymatic Peptides in Bitis arietans Venom Revealed by Comparative Proteomics: Implications for Cross-Reactive Antibody Targeting
Source: Int J Mol Sci. 2026 Jan 31;27(3):1431. doi: 10.3390/ijms27031431 (PMC12898025; doi:10.3390/ijms27031431)
Supplement: Supplementary file 1 [file ijms-27-01431-s001.zip › Supplementary material 6 - Table S3.pdf]

Table S3. Non-enzymatic peptides identified in *Viperidae* venoms.

Peptide ID	Peptide sequence	Class	Subclass	Homologous species	Region type	Found in fractions	Protein accession
N0001	WSDYSSVSYENLVR	C-type lectin	-	*Bothrops*	Surface/binding interface (non-enzymatic toxin)	F2-2:1	Q9PSM6
N0002	GGHLVSIESDEEADFVAQLVAPNIGK	C-type lectin	-	*Bothrops*	Surface/binding interface (non-enzymatic toxin)	F2-2:1	Q9PSM6
N0003	ILEQGEDCDCGSPANCQDR	Disintegrin	-	*Bitis*	Surface/binding interface (non-enzymatic toxin)	F2-2:1	P17497
N0004	GGHLVSIESDEEADFVAQLVAQNIRK	C-type lectin	-	*Bothrops*	Surface/binding interface (non-enzymatic toxin)	F2-2:1	A0A8T1N374
N0005	EFCVELVSDTGYR	C-type lectin	-	*Bothrops*	Surface/binding interface (non-enzymatic toxin)	F2-2:1	A0A8T1N178
N0006	VNYNAWASESECVASK	C-type lectin	-	*Bothrops*	Surface/binding interface (non-	F2-2:1	Q9PSM5

					enzymatic toxin)		
N0007	EGESQMCQALTK	C-type lectin	-	*Bitis*	Surface/binding interface (non-enzymatic toxin)	F2-2:2	Q6T7B5
N0008	EQQCSSEWNDGSK	C-type lectin	-	*Bitis*	Surface/binding interface (non-enzymatic toxin)	F2-2:2	Q6T7B5
N0009	FCMEQANDGHLVSIQSIK	C-type lectin	-	*Bitis*	Surface/binding interface (non-enzymatic toxin)	F2-2:2	Q6X5T3
N0010	KTWEDAEK	C-type lectin	-	*Daboia, Macrovipera, Vipera*	Surface/binding interface (non-enzymatic toxin)	F2-2:3	B4XT05
N0011	IPCAPQDVK	Disintegrin	-	*Bothriechis*	Surface/binding interface (non-enzymatic toxin)	F2-2:3	A0A6B2F6W4
N0012	LYCKDNSPGQNNPCK	Disintegrin	-	*Gloydius*	Surface/binding interface (non-enzymatic toxin)	F2-2:4	Q90Y44
N0013	KIPCAPEDVK	Disintegrin	-	*Bothriechis*	Surface/binding interface (non-enzymatic toxin)	F2-1:2, F2-2:5	A0A6B2F5P9

N0014	IPCAPEDVK	Disintegrin	-	*Bothriechis*	Surface/binding interface (non-enzymatic toxin)	F2-1:2, F2-2:5	A0A6B2F5P9
N0015	KTDLLTR	Disintegrin	-	*Bothriechis*	Surface/binding interface (non-enzymatic toxin)	F2-1:2, F2-2:5	A0A6B2F5P9
N0016	FCVEQAGHLASIESK	C-type lectin	-	*Bitis*	Surface/binding interface (non-enzymatic toxin)	F2-1:1	A0A1B3AXS3
N0017	EEADFVAQLVSENVK	C-type lectin	-	*Bitis*	Surface/binding interface (non-enzymatic toxin)	F2-1:1	A0A1B3AXS3
N0018	CGDDYPFVCK	C-type lectin	-	*Bitis*	Surface/binding interface (non-enzymatic toxin)	F2-1:1	A0A1B3AXS3
N0019	EEADFVAQLISDNIK	C-type lectin	-	*Bitis*	Surface/binding interface (non-enzymatic toxin)	F2-1:1	I7JX23
N0020	DTPFECPSDWSTHRQ	C-type lectin	-	*Bothrops*	Surface/binding interface (non-enzymatic toxin)	F2-1:2	Q9PSM6
N0021	FINSGTICK	Disintegrin	-	*Atheris*	Surface/binding interface (non-	F2-1:9	M5BGY5

					enzymatic toxin)		
N0022	FINSGTICKK	Disintegrin	-	*Atheris*	Surface/binding interface (non-enzymatic toxin)	F2-1:9	M5BGY5
N0023	RGEHCISGPCCR	Disintegrin	-	*Bitis, Cerastes, Echis*	Surface/binding interface (non-enzymatic toxin)	F2-1:9	Q6T6T2
N0024	TMLDGLNDYCTGVTPDCPR	Disintegrin	-	*Bitis*	Surface/binding interface (non-enzymatic toxin)	F2-1:9	Q6T6T2
N0025	EAGEECDCGSPANPCCDAATCKL	Disintegrin	-	*Crotalus*	Surface/binding interface (non-enzymatic toxin)	F3-2:2	P31985
N0026	KPEIQNEIVDLHNSLR	CRISP	-	*Bothrops*	Surface/binding interface (non-enzymatic toxin)	F3-2:2	A0A1L8D673
N0027	YGTGAEPPTAVIGHYTQIVWYK	CRISP	-	*Bothrops*	Surface/binding interface (non-enzymatic toxin)	F3-2:2	A0A1L8D673
N0028	DCPSDWSSYEGHCYRV	C-type lectin	-	*Bothrops, Crotalus, Lachesis, Trimeresurus*	Surface/binding interface (non-enzymatic toxin)	F3-2:2	P0DJC9

N0030	DCPSGWSSYEGHCYRF	C-type lectin	-	*Bothrops*	Surface/binding interface (non-enzymatic toxin)	F3-3:2	P0DJC8
N0031	DCPPDWSSYEGHCYRF	C-type lectin	-	*Bothrops*	Surface/binding interface (non-enzymatic toxin)	F3-3:2	P22030
N0032	AWGSGSQCVASK	C-type lectin	-	*Bothrops*	Surface/binding interface (non-enzymatic toxin)	F3-3:2	A0A2H4N3D3

The table lists peptides mapped to C-type lectins, disintegrins, and cysteine-rich secretory proteins (CRISPs) across *Viperidae* genera. These proteins modulate hemostasis and cell adhesion and contain surface-exposed interaction interfaces, making them potential complementary antigenic targets. Peptides were considered homologous when showing >90% sequence similarity, and preferred accessions were selected using the same criteria described for Table S1.
